# Hospital and Institutional News

**Published:** 1920-07-24

**Authors:** 


### HOSPITAL AND INSTITUTIONAL NEWS.

### THE HARDEST WORKED MAN IN THE KINGDOM.

THE KING AND QUEEN, with Princess Mary, after two weeks spent in the north and the Isle of Man, arrived in North Wales at the end of last week. The first public function attended by the King was when His Majesty opened a sanatorium for the treatment of tuberculous female patients. This institution is at Llangwyfan, near Denbigh, and is the first of a series in Wales which it is hoped will place the principality in the forefront of the fight against consumption. It is a part of the memorial to King George's father, who, as we gratefully remember, was especially interested in, and gave great impetus to the campaign to stamp out the white scourge. His Majesty followed the opening of the Denbigh institution by proceeding the next day to open a similar institution for men at Talgarth, in Mid-Wales, which stands looking south over the Breconshire Beacons and Black Mountains, in a situation most favourable to the treatment of tuberculosis, and amongst most beautiful surroundings. Those who heard the King's reply to the address of welcome at Talgarth, amongst whom were many Welsh listeners, were much pleased by His Majesty's use of the national language on the quotation from an Arthurian legend: "Awake! for it is day. *Deffro, mae'n ddydd.*" The King and Queen, with Princess Mary, who are as hard-worked people it is possible to find on these islands, spent some very full and busy days in Wales, and their programme included a multitude of things, including the inspection of two large metal works near Swansea and the laying of the foundation-stone of the new University College buildings at Singleton, by the foundation of which, to use King George's words, Wales is showing that she seeks the victories of peace as well as those of war.

### A STATE MEDICAL SERVICE.

WE understand that at the forthcoming Trade Union Congress a resolution will probably be put forward calling upon Dr. Addison to provide an improved and extended medical service and hospital treatment under State control. The relative merits of existing medical organisations and a full-time State Medical Service were discussed at considerable length at the recent B.M.A. meeting, and although the latter has, for certain sections of the community, decided advantages, yet the general trend of representative medical and administrative opinion was distinctly against the advisabilty of its adoption at present in this country. In view, however, of the renewed insistence from the Labour Party, the matter will probably come up for further discussion in the near future, and we would therefore remind our readers that in 1918 THE HOSPITAL went very fully into a constructive discussion on the possibilities of a true State Medical Service. The attention then drawn to a number of suggestions based upon a valuable scheme put forward by Col. G. T. K. Maurice, C.M.G., and his very complete outline is now published as "A Vision of State Medical Service" in book form by the Scientific Press, Ltd., Southampton Street, London, price 1s. net.

### SIR JAMES N. DICK.

THE death occurred last week, at Bembridge, Isle of Wight, of Inspector-General of Hospitals and Fleets Sir James Nicholas Dick, K.C.B., who was an honorary surgeon to the King. Sir James Dick was eighty-nine years of age, and served on the active list of the Royal Navy from 1853 to 1891. During the Abyssinian War of 1867-8 he was the senior naval medical officer, and for his services was specially promoted to the rank of fleet surgeon. In the summer of 1868, when in the *Satellite*, he was also the senior medical officer in charge of naval forces and Indian troops in operations on shore against pirates in the Nicobar Islands, Bay of Bengal. Later he was the principal officer at Malta Hospital during the Egyptian campaign and the bombardment of Alexandria, and received from the Admiralty an expression of their high esteem for his zeal, energy, and skill. He was awarded the C.B. in 1887 and the K.C.B. in 1895. On February 25, 1888, he became Director-General of the Medical Department of the Navy. Sir James was the son of Mr. George Dick, formerly a lieutenant in the 75th Regiment, and he married, in 1868, Elizabeth, daughter of Mr. Robert Beveridge. For twenty-seven years he had been an Honorary Surgeon to the Sovereign, having been first appointed under Queen Victoria in 1893.

### FEDERATION OF MEDICAL AND ALLIED SOCIETIES.

ON Tuesday, July 27, 1920, will be held at the Council-room, 11 Chandos Street, Cavendish Square, W., the annual meeting of the above Federation. The general meeting will be at 4 P.M., following the annual business meeting, held one hour earlier. A review of the work of the Federation will be considered, and also the need for constructive effort by the medical and allied professions in the organisation of the medical services of the country. Among the speakers will be Lord Dawson of Penn and Captain W. E. Elliott, M.P. In the past year, among other activities of the Federation, have been carried out a series of inquiries among societies co-operating regarding a possible medical service under the Health Ministry and upon the possibilities of a Pure Milk Bill. Also an exhaustive consideration was commenced in December 1919 of the National Health Insurance Benefit Regulations for 1920. A large number of societies have been supported in their work, including the British Dental Association, the British Science Guild, the Medico-Psychological Association, the College of Nursing, etc., and in each case we appreciate the real service whch the Executive Council has been able to render. The necessary legal procedure in regard to incorporation of the Federation is well forward, and should be completed at an early date.

### DR. ADDISON AND THE VOLUNTARY HOSPITALS.

The Minister of Health, in a recent debate on supply, made it clear that his department is not yearning for the control of voluntary hospitals. He was as usual, cryptic as to the extent to which such institutions would enter into any general health scheme, but he stated in plain words that the Ministry of Health have enough responsibility in the administration of their own services to save them from any plan of State control. He paid the hospitals a compliment in acknowledging that they are pioneers of efficient service, in whose regard it would be a mistake of the first order to interfere with that efficiency, and any scheme that will be put forward will be to promote their public utility. The Minister announces that a survey of Poor-Law hospitals from a medical point of view has been arranged in order that a complete understanding may be brought about as to the extent of the hospital services, voluntary and Poor-Law, available at the present time.

### "THE CLINICAL JOURNAL."

We have received a copy of the *Clinical Journal*, which is now again to be issued weekly, and we are glad to note the revival of this very useful periodical. Founded in 1892, the object has been specially to report and publish clinical lectures given by the leading physicians and surgeons of the day—lectures for the most part delivered to a handful of students and quite lost to the majority of general practitioners. Their usefulness in helping to keep the working doctor abreast with all current medical progress is obvious, and we wish the journal every success.

### A CURE FOR LEPROSY.

Although the details to hand are not complete, there is apparently considerable truth in the recent claim that the active principle of chaulmoogra oil, the only therapeutic agent accepted as valuable in leprosy cases, has been isolated by workers in the Hawaiian Islands leper colony. The great point of interest lies in the fact that by isolating the medically active portion of the drug the healing power and drug action can be standardised, and a treatment scheme devised whereby its administration can be made continuously and until a cure is affected. The local health service is now conducting a careful study of the possible methods in which the chaulmoogra principle may best be used, making records of every case, photographing lesions monthly, and carrying out the whole investigation in a thoroughly businesslike manner. To their collected results, which will be published later, we look forward with great interest.

### SALARIES AND THE CIVIL SERVICE AWARD.

Considerable dissatisfaction has arisen in the public services regarding the present financial position of the heads of departments. The last two or three years has seen the salaries of the lower-grade men gradually rise, whilst their chiefs are receiving the same amounts as in pre-war days. There is a general antipathy to increase the salaries of the highly-paid officers, though under the award of the Civil Service they are entitled to substantial increases. A sanitary inspector whose basic rate of salary was £300 is now entitled to receive under this award £528, and has received it; but his medical officer, whose salary is £1,100, and who is entitled to an increase of £154, does not get it. But while the heads of departments have just cause for grievance, many of those under them have similar grounds for complaint. Public authorities, more especially Boards of Guardians, will not apply this scale to their employees, and the consequence is great unrest. Mr. Frank Briant, M.P., has warned his colleagues at Lambeth Board of Guardians that they were not acting wisely in an endeavour to postpone this question for three months. Fortunately, they took his advice, and will attempt to solve the problem at their next meeting, instead of staving off the day when the employees both in their infirmaries and institutions will receive the money to which they are entitled under the Civil Service award.

### TAX-FREE BENEFACTIONS.

Mr. Basil Mayhew, in a letter to *The Times*, revives the subject of the possibility of the Government making an allowance in respect of income tax to persons who are willing to provide funds for charitable purposes. He makes no new contribution to the subject which is likely to be helpful to the Chancellor of the Exchequer, except that such relief should be confined to hospitals, on the ground that, for the moment at any rate, they are in a class apart from other charities and worthy of special consideration. We dealt with this matter in our issue of July 10, page 386, when the reasons for and against such a system were fully detailed. In the debate on the excess profits duty in the House of Commons last week Mr. Chamberlain announced a concession which, although not extensive, is of considerable interest. He promised to include in the Finance Act a provision whereby out of the profits of a trade or business any contribution has been made after July 16, 1920, to any trust, society, or body of persons in the United Kingdom, established solely for the purpose of the relief of the poor or the sick, or for the advancement of education, or for scientific research, there shall for the purpose of excess profits duty be allowed for the period covered an amount not exceeding five per cent. of the excess profits, before adjustment on account of capital, and before other statutory deduction, and not exceeding twenty per cent. of the amount of such contribution. This concession is apart from existing allowances with regard to local institutions, etc., already provided for as representing trading expense. The concession establishes a principle, although the sums involved will, except in but a few instances, be small. If a person or firm, for instance, should be paying excess profits duty on £10,000, only £100 would be the legitimate deduction under the new section; but there are, of course, cases where much larger sums are involved, and it remains to be seen in what way the incidence of the Chancellor's new provision will fall upon the finances of voluntary hospitals.

### REGISTRATION WITHOUT EXAMINATION.

The first list of persons accepted for registration as pharmacists (chemists and druggists) has now been published, and comprises dispensers, all of whom have been engaged under certain conditions at approved institutions for seven years, prior to the passing of the by-law entitling them to registration. Much opposition was shown when the Pharmaceutical Society, under outside pressure, agreed to form a by-law last year, but by a majority vote it was decided to accept certain persons under stringent conditions, which ensured that the degree of knowledge they possessed warranted registration. Whilst the principle of registration without examination is unsound, yet other professions are experiencing similar conditions, notably dentistry, in which it is proposed to register as "dentists" unqualified persons who have undergone no curriculum nor passed any of the recognised examinations as in the case of duly qualified "dental surgeons."

### THE KING AND WAR MEMORIALS.

The King was invited by the Southwark Board of Guardians to unveil the war memorial at their infirmary, in memory of those who died during the great war, at the Southwark Military Hospital, when the institution was used by the War Office. His Majesty's private secretary has informed the Guardians that the King is unable to accede to the request, as he does not unveil local memorials, but only those of a national character. Miss Gow, the Chairman of the Infirmary Visiting Committee, who for a great many years has been associated with work amongst the poor of Southwark, will unveil the memorial when it is completed.

### TWO IMPORTANT SECRETARIAL APPOINTMENTS.

The vacancy in the office of secretary to the Bristol Royal Infirmary, which was announced in our pages at the end of June, has been filled by the appointment of Mr. E. C. Smith, the present secretary and general superintendent of Queen's Hospital, Birmingham. Mr. Smith's hospital experience extends over twenty years, beginning at the Swansea General and Eye Hospital, where he was for ten years assistant secretary. From there he went as secretary to the North Ormesby Hospital, Middlesbrough, where he remained six years, and in 1914 he succeeded Mr. Arthur Hulme at Birmingham. The work at Bristol should give him plenty of scope and we shall watch his progress with interest. The appointment of a secretary to St. Mary's Hospital, Paddington, in succession to Mr. Thomas Ryan who is retiring, has also been made during the past week. There were 400 candidates for this appointment. The selection has fallen on Mr. Walter Parkes, an officer in the Education Department of the London County Council, who, though possessing administrative experience and ability, has not had previous hospital experience. Mr. Parkes has been in the service of the L.C.C. for some seven or eight years, part of which were spent in the Army during the war, where he gained distinction as a gallant and capable soldier and officer. He has since been seconded by the L.C.C. to the Ministry of Labour in connection with its education schemes for discharged disabled men. Mr. Parkes, who is thirty-two years of age, will take up his duties at St. Mary's in October, and till the end of the year or possibly later will have the advantage of Mr. Ryan's assistance in an advisory capacity.

### THE M.A.B. AND THIRD LONDON GENERAL HOSPITAL.

Recent references were made in these columns to the fact that the buildings and equipment of the Third London General Hospital are for sale, and that there was a danger of this very valuable establishment being broken up and distributed. The latest news is that the Metropolitan Asylums Board are considering if it is possible for them to take over the hospital and work it within their system. An important point which has not hitherto been brought out is that these temporary buildings stand upon what is common land, and we have many instances before us of the difficulty the public experience in regaining the enjoyment of those parts of open spaces which were taken from them for the purposes of the war, under the Defence of the Realm Act. Whoever, therefore, takes possession of the Third London should do so on the clear understanding that the tenure is of strictly limited extent. As a matter of economy, on the other hand, it seems reasonable that what would cost something like £100,000 to provide, but would realise only £8,000, should be made use of for a few more years.

### HOSPITALS AND MISSIONS.

A correspondence in the *Globe* has led to an interesting statement from a missionary, who says that the supporters of foreign missions are the people who are most generous to hospitals at home. Since the proportion of those who give to charity is small, the statement is probably correct, and gathers weight from the fact that most foreign missions have hospitals attached to them. We have often urged that all foreign missions should be medical missions and that a medical training should be part of the equipment of every candidate for mission work in the East. This leads us, again, to suggest that the foreign missions would interest the home public more than they do if they gave more news of the medical and hospital side of their work, which is always read eagerly by hospital workers in England. Another effect of such frankness would be to attract trained nurses, who are often willing to travel and to live abroad if a suitable opportunity for carrying on their profession is offered to them. Why are the missionary societies so slow to impart information?

### MENTAL HOSPITAL BUILDING STOPPED.

The Board of Control has led the Croydon Mental Hospital authorities—that is, the Town Council—to abandon the extensions to the institution, agreed upon so long ago as May 1915. The reason is that the vacation of a number of mental hospitals by the military is deemed sufficient for boarded-out patients at a time when the cost of living is so high. The agreement with the architect, Mr. H. Carter Pegg, has therefore been determined, and under its terms he will be paid for his services to the date of the determination.

### A SCOTCH HOSPITAL SABBATH.

The Young Men's Guild of the Church of Scotland lately proposed to gather the younger members of the different churches at a Covenanting and Memorial Service in the churchyard of Greyfriars, where conscience-troubled folk in an earlier age were imprisoned, and to have a collection for the Edinburgh Royal Infirmary. The Band of Comrades of the Great War was to play, and the choirs of all the churches to sing. Such an effort is welcome, for the Edinburgh hospitals groan for want of money. Besides, Hospital Sunday is difficult to organise because, unlike the Saturday Fund, it has no methodical source of collection, and is largely dependent upon the fluctuating habit of church-going.

### LORD ARMSTRONG'S ASSURANCE.

Two interesting statements were made at the recent quarterly court of the Royal Victoria Infirmary, Newcastle. Mr. G. Ollier, vice-chairman of the finance committee, reported a deficit of over £6,000 for the past quarter, but said that the North Country had come to the hospital's help. Mr. Ollier said that he did not want State control, but that "after we have done our best to make ends meet, the Government should step in and annul any remaining deficit." That view has its advocates to-day, but it is their obligation to prove that such fluctuating assistance can be so safe-guarded as not to interrupt voluntary subscriptions. The State, at all events, is not eager to shoulder the burden. In fact the Ministry of Health seems more coy than most of the Departments to spend the public money. Glad that this is so, we are glad also that Lord Armstrong, who spoke at the same meeting, was able to say that the income of the hospital had already increased for the year by £10,000; and that he hoped before the end of the year that the hospital would nearly be able to meet its expenses.

### THE NEW ST. LUKE'S HOSPITAL.

We are informed that the arrangements for rebuilding St. Luke's Hospital for Mental Diseases, Old Street, E.C., which has been closed since the end of 1916, are making good progress. Plans for rebuilding the first part of the new St. Luke's Hospital, on the "Welders" Estate, near Gerrard's Cross, Bucks, are practically completed, and the Committee is hopeful that they will shortly receive the approval of the Board of Control and the other public authorities concerned, so that building operations may begin this year, or certainly in the early part of 1921. The work of reconstruction has been considerably delayed, owing to the vastly increased costs, combined with a shortage of building materials. In these circumstances the Committee has wisely decided to rebuild the institution in sections as opportunity and funds will permit.

### BACKYARD BEDS FOR CONSUMPTIVES.

Southwark Borough Council's Health Committee, after considering the provision of shelters for consumptive patients likely to benefit by sleeping out of doors, reports that the type of shelter needed would be small and movable for use in small backyards and gardens. The tuberculosis officer has gone into the whole matter and reported upon a type of small shelter which he considers would be most suitable in the limited accommodation usually available. The cost of such shelters is about £12 10s. each, and it is understood that half the cost would be borne by the Ministry of Health. At present the Committee merely suggests experimenting with the proposal, but that the free provision of these shelters to consumptives should be a policy considered by the Council.

### INFECTIOUS HOSPITAL, POONA.

The old corrugated-iron structure known as the Infectious Diseases Hospital will shortly give place to a permanent structure more in keeping with the requirements of a large station like Poona. The Government has acquired land for the use of the new hospital. Remembering the fearful epidemics of plague Poona has passed through ever since the fateful December of 1896, when the disease first broke out in the city, the part played by the infectious hospitals has been noteworthy. The place was run up, shed by shed, writes a correspondent, as rapidly as possible. But the disease always outstripped the accommodation. The sheds that did duty during the opening years of the present century are still standing, but the chief buildings have been put to use for other diseases, such as smallpox and cholera. The Government is now going to give the hospital permanent form on the present site. The Sassoon Hospital does not take in patients suffering from infectious diseases, and these have to go to a quasi-temporary hospital.

### THIS WEEK'S DRUG MARKET.

The position is much the same as it has been for the past few weeks; buyers still hold off and the easier tendency of prices is strongly in evidence. To tempt purchasers of large quantities of drugs considerable concessions might have to be made, and prices at present quoted are in many cases merely nominal. There is no considerable change in the values of synthetic drugs generally, but salicylic acid is offered at lower prices and the same applies to aspirin, while the general price tendency of products of this class is downwards. Potassium bromide is offered at irregular prices and there is a plentiful supply; the demand for sodium, and ammonium bromides is rather better. Citric acid is cheaper. Camphor and menthol are both quiet, and prices still have a sagging tendency. Cocaine is in better supply and offered at rather lower figures. Cod-liver oil is again lower, but buyers still hold aloof in the hope that prices will recede still further in face of the plentiful supplies. A substantial fall in the price of ergot of rye is anticipated.

